# An ex vivo continuous passive motion model in a porcine knee for assessing primary stability of cell-free collagen gel plugs

**DOI:** 10.1186/1471-2474-11-283

**Published:** 2010-12-15

**Authors:** Turgay Efe, Markus D Schofer, Alexander Füglein, Nina Timmesfeld, Susanne Fuchs-Winkelmann, Thomas Stein, Bilal Farouk El-Zayat, Jürgen RJ Paletta, Thomas J Heyse

**Affiliations:** 1 Department of Orthopedics and Rheumatology, University Hospital Marburg, Baldingerstrasse, 35043 Marburg, Germany; 2 Department of Oral and Maxillofacial Surgery, University Hospital Aachen, Pauwelsstrasse 30, 52074 Aachen, Germany; 3 Institute of Medical Biometry and Epidemiology, Philipps-University Marburg, Bunsenstrasse 3, 35037 Marburg, Germany

## Abstract

**Background:**

Primary stability of cartilage repair constructs is of the utmost importance in the clinical setting but few continuous passive motion (CPM) models are available. Our study aimed to establish a novel ex vivo CPM animal model and to evaluate the required motion cycles for testing the mechanical properties of a new cell-free collagen type I gel plug (CaReS^®^-1S).

**Methods:**

A novel ex vivo CPM device was developed. Full-thickness cartilage defects (11 mm diameter by 6 mm deep) were created on the medial femoral condyle of porcine knee specimens. CaReS^®^-1S was implanted in 16 animals and each knee underwent continuous passive motion. After 0, 2000, 4000, 6000, and 8000 motions, standardized digital pictures of the grafts were taken, focusing on the worn surfaces. The percentage of worn surface on the total CaReS^®^-1S surface was evaluated with image processing software.

**Results:**

Significant differences in the worn surface were recorded between 0 and 2000 motion cycles (p < 0.0001). After 2000 motion cycles, there was no significant difference. No total delamination of CaReS^®^-1S with an empty defect site was recorded.

**Conclusion:**

The ex vivo CPM animal model is appropriate in investigating CaReS^®^-1S durability under continuous passive motion. 2000 motion cycles appear adequate to assess the primary stability of type I collagen gels used to repair focal chondral defects.

## Background

Cartilage defects of the knee are commonly encountered in orthopedic clinical practice [1,2]. Several well described repair strategies have been used in the management of chondral lesions [3-6]. Debate persists about the best repair technique for symptomatic chondral defects of less than 2 cm² [7]. Currently, CaReS^®^-1S (Arthro Kinetics, Esslingen, Germany), a cell-free collagen type I gel plug was introduced to treat isolated cartilage defects. This graft is implanted in the debrided chondral lesion, in a single-stage procedure. The gel triggers chondrocyte migration and proliferation into the plug, which has been proven both in vitro and ex vivo [8].

Most cartilage regenerative studies address the biochemical and histological composition of generated cartilage [9,10]. However, successful cartilage repair requires sufficient initial mechanical stability of the grafts [11]. The implanted grafts should withstand the forces in vivo in the early postoperative phase during joint movement. Secure primary stability is necessary to keep the grafts in place and to achieve sufficient joint surface congruity, on which the long-term results depend [12]. Partially or completely delaminated tissue engineered constructs can cause locking in the knee and poor clinical results [13,14].

Several authors describe the beneficial effects of continuous passive motion (CPM) on neochondrogenesis and most postoperative protocols consider ex vivo non-weight bearing CPM in the first 6 weeks after surgery to be beneficial [15-17].

Drobnic et al. [18] reported a human cadaveric model of ex vivo CPM, testing four fixation techniques for fibrinogen and thrombin coated collagen fleece. They aimed to simulate the initial postoperative period by applying ex vivo CPM to specimens. However, the availability of appropriate human specimens without advanced osteoarthritis is limited. This can be a challenge for developing appropriate protocols to test the primary stability of collagen plugs; to date, there are no established animal models.

Moreover, little is known about the required motion cycles for testing the mechanical properties of cartilage repair constructs. The means to assess the early postoperative phase after cartilage regenerative procedures is lacking. Consequently, efforts to build a new ex vivo CPM device to assess the mechanical properties of tissue engineered scaffolds would be of great value. The aim of the present study is to construct a simplified, custom-made ex vivo CPM device and to investigate the number of motion cycles required for testing the mechanical properties of collagen plugs. We hypothesized that the new device would allow us to reliably evaluate the degree of wear to collagen plugs. We also aimed to determine the number of cycles required to assess wear appropriately using this model.

## Methods

Sixteen porcine knees (age 9 months) were used to assess primary stability of cell-free collagen gel plugs. These were obtained from the local butcher, fresh frozen at -25°, and thawed for 16 hours at room temperature (20°C) before testing. This project was performed in accordance with the Helsinki Declaration and with local legislation. CaReS^®^-1S was implanted in an identical manner in each specimen. Right and left knees were not differentiated; both knees were used [19]. Some knee specimens were dissected to evaluate the anatomy of the porcine knee and to establish the surgical procedure.

### CaReS^®^-1S

CaReS^®^-1S is a sterile cylindrical, three-dimensional collagen-based and cell-free gel plug, consisting of 4.8 mg/mL rat tail collagen type I (CaReS^® ^[20,21], Arthro Kinetics, Esslingen, Germany). It has been used for cartilage defects in the knee and ankle in patients under 50 years of age. The plug is available in three different diameters (11, 22, and 34 mm) and thicknesses (4, 6, and 8 mm). The hydrogel consistency allows adequate coverage and sufficient reconstruction of the articular surface. The basic concept of CaReS^®^-1S is to replenish the cartilage defect with a matrix structure, facilitating autologous chondrocytes to penetrate into the lesion zone. Low antigenicity and high biocompatibility of CaReS^®^-1S is achieved by the high conservation of protein sequences of collagen type I within various species. The plugs used in this study measured 11 mm diameter by 6 mm deep and contained 4.27 mg collagen type I. CaReS^®^-1S were stored in phosphate-buffered saline solution and preserved at 4°C until use.

### Ex vivo CPM device

**Figure 1:**
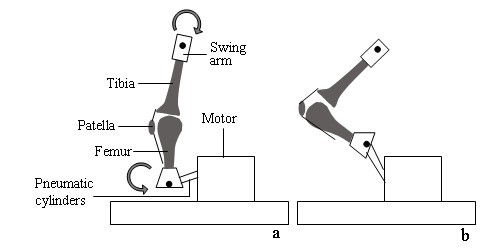
**Schematic diagram of the new ex vivo CPM device in extension (a) and flexion (b)**.

A custom-made, pneumatic ex vivo CPM device was constructed, on which extension-flexion motions were performed at a frequency of 1 Hz (Figure 1). There were no constrained forces and during passive motion the tibia was allowed to rotate freely. To simulate early postoperative rehabilitation, an additional axial load during testing was not performed. The extension and flexion motion cycles was performed via the coordination of two pneumatic cylinders and detected by an integrated counter.

### Surgical procedure

The tibia and the femur were transected approximately 10 cm proximal and distal to the knee joint, preserving muscles, articular capsule, ligaments, and tendons. A medial parapatellar arthrotomy was performed and the patella was dislocated laterally. A standardized full-thickness cartilage defect of 11 mm diameter by 6 mm deep was created in the weight-bearing area of the medial femoral condyle using a punch (Arthro Kinetics, Esslingen, Germany).

### Ex vivo CPM protocol

**Figure 2:**
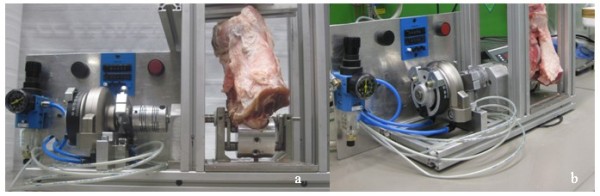
**Experimental setup with the **ex vivo **CPM device and fixed porcine knee specimen in front (a) and side (b) views**.

Experiments were performed at 20°C. A constant ex vivo CPM protocol was established for the performance of experiments. After the preparation of the chondral defect, the proximal femur and distal tibia were fixed into a cylindric metal device and fastened in the vertical position (Figure 2). As the porcine hind leg could not be fully extended, specimens were flexed to and from an extension position, between 20°-120°-20°, encompassing one complete motion cycle. After fixation, ex vivo CPM device test cycles through the whole range of motion were performed to align the mechanical axis of the stifle joint with the mechanical axis of the ex vivo CPM device. Both tibial and femoral fixation allowed adjustment of the stifle position. After adjustment, 0.3 mL of a two-component fibrin sealant (Tissucol Duo, Baxter, Unterschleißheim, Germany) was applied to the prepared defect and the surrounding cartilage rim, prior to CaReS^®^-1S placement into the lesion. The gel plug was pressed into the defect according to standard manufacturer guidelines and clinical practice. The graft was considered to be sufficient when complete congruity with the surrounding cartilage rim was achieved. 5 mL of 0.9% sodium chloride solution was injected intraarticularly with a catheter to keep the articular surface moist and intraarticular friction low. After 2000, 4000, 6000, and 8000 motion cycles, the arthrotomy was reopened to evaluate the graft worn surface and to moisten the joint surface with 0.9% sodium chloride solution. Standardized digital photographs were taken for analysis after CaReS^®^-1S implantation and after implantation and every 2000 motion cycles. During the entire procedure, test specimens were externally moistened with 0.9% sodium chloride solution every 10 minutes.

### Graft Evaluation

**Figure 3:**
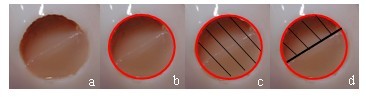
**Worn surface analysis of CaReS^®^-1S**. (a) initial image; calculation of percentage of worn surface from the ratio of (c) and (d).

The surface damage was defined as intact, marginally detached, partially detached, or completely displaced. Marginally detached only involved the junction between CaReS^®^-1S and adjacent cartilage, and appeared as a fissure. In partial detachment, the graft only covered a part of the defect. If the cartilage defect was completely empty, this was defined as displacement of the graft. Digital photographs obtained after 0, 2000, 4000, 6000, and 8000 motion cycles were transferred using image processing software (QUIPS, Leica, Wetzlar, Germany). The worn surface area and the total area of the defect were measured in pixels; the percentage of worn surface was calculated from the ratio of both measurements (Figure 3). The areas of interest were traced by an independent observer who was not involved in performing the experiments.

### Statistical analysis

The repeated measured data at 2000, 4000, 6000, and 8000 motion cycles were assessed by analysis of variance (ANOVA). The increase in the worn surface within the first 2000 motion cycles and differences between 2000, 4000, 6000, and 8000 motion cycles were analyzed through the application of general linear hypothesis testing. P-value adjustment was performed by the method of Shaffer [22]. Additionally, for all contrasts, the corresponding 95% family-wise confidence intervals (CI) were calculated. Two-sided adjusted p-values less than 0.05 were considered statistically significant. All statistical analysis was performed with R software (Foundation for Statistical Computing, Vienna, Austria).

## Results

**Figure 4:**
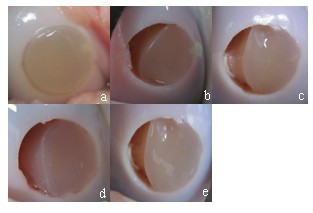
**CaReS^®^-1S surface at 0 (a), 2000 (b), 4000 (c), 6000 (d), and 8000 (e) motion cycles**.

The ex vivo CPM device worked for 8000 motion cycles. The most time-consuming procedure was the mechanical axis of the stifle joint alignment with the mechanical axis of the testing device. Within the first 2000 motion cycles, the mean worn surface significantly increased by 20.2% (p < 0.0001, C.I. 10.2% - 30.2%). After 2000 cycles, no significant changes in the worn surface were observed (Table 1). Representative photos at 0, 2000, 4000, 6000, and 8000 motion cycles are shown in Figure 4. The evaluation during ex vivo CPM showed that none of the CaReS^®^-1S grafts delaminated totally with an empty defect site.

**Table 1:** Analysis of surface wear in dependence of motion cycles (p < 0.05)

Motion Cycles	Mean difference of worn surface (%)	95% Confidence Interval	P-Value
0 to 2000	20.2	10.2 - 30.2	0.0001
2000 to 4000	1.9	-1.5 - 5.3	0.20
2000 to 6000	2.9	-0.6 - 6.3	0.12
2000 to 8000	2.3	-1.1 - 5.7	0.20

## Discussion

Information on ex vivo CPM devices and the number of motion cycles needed to investigate cartilage repair constructs is lacking. The aim of our study was to construct a novel ex vivo CPM device and to create a small animal model to evaluate the required number of continuous passive motions for testing CaReS^®^-1S. Our hypothesis that this device would allow reproducible evaluation of wear to collagen plugs was proven to be correct. We also successfully determined number of cycles required to assess wear appropriately.

There are several limitations to our study. While porcine anatomy closely approximates human anatomy [23], immanent differences between species are evident. Mechanical in vitro studies only simulate the physiological function and motion of the joint, since there is no force applied by the muscles. The presented ex vivo CPM device may only partially simulate its clinical application. In our study, analysis of the worn surface of the graft was performed by imaging processing software as a semiquantitative analysis. More objective quantitative measurements and absolute values may be preferable. However, analysis of cartilage repair constructs with the ex vivo CPM is difficult, raising the question of reliability. Other studies used qualitative scales [18,24], posing the problem of subjective bias. It is very likely that a potential malalignment of the joint within the testing device may have affected the results. In our setup, the tibia was allowed to rotate freely. It was also allowed to move freely in the frontal plane. Malalignment in the testing device resulted in impingement of the specimen with the frame. Simulation in human cadaver knees would be the optimal standard for testing. However, human specimens are expensive, sometimes difficult to obtain, and come from elderly people. Therefore, osteoarthritic changes are a common finding in these specimens, which may interfere with the evaluation of a procedure in an otherwise healthy joint of a young patient. The porcine model comes with the advantage of unlimited availability at a low cost.

There are currently only two experimental ex vivo CPM models for testing cartilage implants. A recent study on human cadaver knees investigated the quality of PEOT/PBT scaffold fixation (transosseous fixation, fibrin glue, biodegradable pins, and continuous cartilage sutures) by ex vivo CPM in a vertical position [24]. The mechanical behavior was evaluated after 60 and 150 motion cycles with a 35N load, focusing on outline attachment, scaffold integrity, and area coverage. After 210 motion cycles, an endpoint fixation test was performed. Drobnic et al. [18]compared in a similar model four different fixation techniques (self-adhesion, fibrin glue, bone sutures, periosteal cover) of a collagen scaffold in the human cadaveric knee, horizontally oriented with and without loading. In both studies, the numbers of cycles remained small and did not correspond with the clinical situation after surgical management of cartilage defects; whereas ex vivo CPM is often applied several hours a day. It is most likely that in the early postoperative phase the knee undergoes considerably more than 150 motion cycles. At our institution and in previous experience [25] patients usually use the ex vivo CPM machine for 6 to 8 hours daily after implantation of tissue engineered constructs. The rate is one cycle per minute but varies based on patient comfort and performance. This fact should be taken into consideration when performing mechanical testing. A study design with fewer motion cycles makes the transformation from in vitro to in vivo testing difficult. Consequently, long-term durability studies are necessary. However, it is possible to determine if such constructs were too fragile to be practical. Thus, shorter-term motion cycles studies have their place. In the present study, testing of CaReS^®^-1S was performed with 8000 motion cycles under ex vivo CPM. The question of how many motion cycles were necessary to evaluate wear to collagen plugs has not been answered satisfactorily as of yet. In our study, significant changes in the worn surface of the graft were only detected between 0 and 2000 motion cycles (p = 0.0001). Therefore, a protocol with 2000 motion cycles appears to be a sufficient number to investigate type I collagen gels used to repair focal chondral defects.

Delamination of tissue engineered constructs occurs mainly in the first six postoperative months[26]. A possible explanation for this may include weak mechanical fixation and adherence. Since it is known that friction forces and shear forces may cause delamination of cartilage implants [27], most authors allow only partial weight-bearing after cartilage regeneration techniques [28,29]. While Drobnic et al. [18] and Bekkers et al. [24] used additional load ex vivo CPM protocols, intraarticular pressure measurements were not performed. It remains unclear how much load was applied on the specimens intraarticularly. Although no additional load was applied in our study, we assume that the vertical orientation, the corresponding tibial cartilage, and the intraarticular liquid generates friction forces and shear forces on the gel that correspond with the clinical setting. Nevertheless, performing the experiments with an additional load might have been preferable.

Evaluation of tissue engineered constructs in the early postoperative rehabilitation phase is difficult. The ex vivo CPM device used in the present study is simplified and can be helpful to investigate the durability of collagen gel constructs. Our findings may support research in new materials in a porcine knee model. Nevertheless, the ex vivo CPM device cannot be used in assessing biomechanical properties (viscoelasticity, permeability, tensile strength, and electromechanical properties).

## Conclusion

The novel custom-made ex vivo CPM device allows the investigation of CaReS^®^-1S under a high number of motion cycles. Significant differences in the worn surface were detected between 0 and 2000 motion cycles, but not beyond 2000 cycles. This device and study model may be of great value in further investigations.

## Competing interests

TE, MDS, BFE and TJH are consultants to Smith&Nephew.

## Authors' contributions

TE participated in the study design, carried out the study, interpreted the results and drafted the manuscript. MDS, SFW, and TS participated in the study design and interpretation of the results. AF constructed the ex vivo CPM device and participated in the study design. NT performed the statistical analysis. JRJP and BFE designed the protocol and helped with data interpretation. TJH participated in the study design and the drafting of the manuscript. All authors read and approved the final manuscript.

## Pre-publication history

The pre-publication history for this paper can be accessed here:

http://www.biomedcentral.com/1471-2474/11/283/prepub
